# The Prognostic Impact of Circulating Regulatory T Lymphocytes on Mortality in Patients with Ischemic Heart Failure with Reduced Ejection Fraction

**DOI:** 10.1155/2020/6079713

**Published:** 2020-02-10

**Authors:** Andreas Hammer, Patrick Sulzgruber, Lorenz Koller, Niema Kazem, Felix Hofer, Bernhard Richter, Steffen Blum, Martin Hülsmann, Johann Wojta, Alexander Niessner

**Affiliations:** ^1^Division of Cardiology, Department of Internal Medicine II, Medical University of Vienna, Austria; ^2^Ludwig Boltzmann Cluster for Cardiovascular Research, Vienna, Austria

## Abstract

**Background:**

Heart failure with reduced ejection fraction (HFrEF) constitutes a global health issue. While proinflammatory cytokines proved to have a pivotal role in the development and progression of HFrEF, less attention has been paid to the cellular immunity. Regulatory T lymphocytes (Tregs) seem to have an important role in the induction and maintenance of immune homeostasis. Therefore, we aimed to investigate the impact of Tregs on the outcome in HFrEF.

**Methods:**

We prospectively enrolled 112 patients with HFrEF and performed flow cytometry for cell phenotyping. Individuals were stratified in ischemic (iHFrEF, *n* = 57) and nonischemic etiology (niHFrEF, *n* = 57) and nonischemic etiology (niHFrEF,

**Results:**

Comparing patients with iHFrEF to niHFrEF, we found a significantly lower fraction of Tregs within lymphocytes in the ischemic subgroup (0.42% vs. 0.56%; *p* = 0.009). After a mean follow-up time of 4.5 years, 32 (28.6%) patients died due to cardiovascular causes. We found that Tregs were significantly associated with cardiovascular survival in the entire study cohort with an adjusted HR per one standard deviation (1-SD) of 0.60 (95% CI: 0.39-0.92; *p* = 0.009). After a mean follow-up time of 4.5 years, 32 (28.6%) patients died due to cardiovascular causes. We found that Tregs were significantly associated with cardiovascular survival in the entire study cohort with an adjusted HR per one standard deviation (1-SD) of 0.60 (95% CI: 0.39-0.92; *p* = 0.009). After a mean follow-up time of 4.5 years, 32 (28.6%) patients died due to cardiovascular causes. We found that Tregs were significantly associated with cardiovascular survival in the entire study cohort with an adjusted HR per one standard deviation (1-SD) of 0.60 (95% CI: 0.39-0.92; *p* = 0.009). After a mean follow-up time of 4.5 years, 32 (28.6%) patients died due to cardiovascular causes. We found that Tregs were significantly associated with cardiovascular survival in the entire study cohort with an adjusted HR per one standard deviation (1-SD) of 0.60 (95% CI: 0.39-0.92;

**Conclusion:**

Our results indicate a potential influence of Tregs in the pathogenesis and progression of iHFrEF, fostering the implication of cellular immunity in iHFrEF pathophysiology and proving Tregs as a predictor for long-term survival among iHFrEF patients. A preview of this study has been presented at a meeting of the European Society of Cardiology earlier this year.

## 1. Introduction

Hearth failure with reduced ejection fraction (HFrEF) represents a major health issue in western-industrialized countries with an estimated prevalence of more than 37 million people worldwide [1]. As multifaceted syndrome caused by structural damage and subsequent dysfunction of the cardiac tissue, it is mainly promoted via inflammatory response triggered by cardiac tissue damage and functional remodeling [2–5]. In this regard, there has been growing evidence of close pathophysiological links between the progression of HFrEF and both local and peripheral proinflammatory states. Inflammatory biomarkers such as high-sensitivity C-reactive protein (hsCRP) or cytokines were found to have a major predictive potential on the development and progression of HFrEF. However, while data on peripheral cytokines has been widely investigated, less attention has been paid to the association of cellular immunity. In this regard, T lymphocytes were recently found among cardiac tissue in both patients with ischemic and nonischemic HFrEF—suggesting their importance in the modulation of cardiac remodeling driven by inflammatory stimuli [6, 7]. It is well investigated that exaggerated immune activity is closely related to HFrEF severity and patient outcome by an increase of proinflammatory cytokines and both activation and recruitment of several T cell lines [8–10]. Among these, intrinsic proinflammatory stimuli, mostly TNF-alpha and its involvement in cardiac remodeling through the recruitment of further—especially cytotoxic—T cell populations, have recently been identified as an independent predictor for outcome in HFrEF individuals [11]. As a major modulator of the inflammatory response, regulatory T cells (Tregs) induce and maintain immune homeostasis through TGF-b, IL-2, and IL-10. It consequently suppresses T cell activation, proliferation, and cytokine production and therefore inhibits an exaggerated immune response and most importantly potentially unjustified tissue damage and cardiac fibrosis. Considering the strong association of T lymphocytes and outcome in HFrEF, it seems intuitive that regulatory Tregs might have a major impact on the limitation of inflammation-triggered local tissue damage, cardiac fibrosis, and subsequently the outcome of patients at risk. Therefore, we aimed to investigate the impact of regulatory T cells on patient outcome in HFrEF.

## 2. Methods

### 2.1. Study Population

Within the present investigation, we prospectively enrolled 112 patients with HFrEF between January 2008 and December 2010 at a specialized outpatient department for the management of heart failure of the Medical University of Vienna, Department of Cardiology (Austria). All patients received an optimal and personalized medical treatment approach in accordance to the latest guidelines of the European Society of Cardiology (ESC). The presence of HFrEF was defined in accordance to the current guidelines of the ESC for the management of heart failure: New York Heart Association functional classification (NYHA) ≥ II and either left ventricular ejection fraction (LVEF) < 40% and/or N-terminal pro B-type natriuretic peptide (NT-proBNP) values > 500 pg/ml. Ischemic HFrEF was classified as acquired heart failure based on significant coronary vessel disease and/or prior acute myocardial infarction. Patients presenting with any kind of inflammatory conditions, active infections, autoimmune diseases, or malignancies were not eligible for study inclusion. The total study population was stratified into two subgroups according to etiology—ischemic (iHFrEF, *n* = 57) and nonischemic etiology (niHFrEF, *n* = 55). Peripheral venous blood samples of all 112 subjects were available for flow cytometry analysis. Participants were followed prospectively until December 2014 or until the primary endpoint was reached. No patient was lost during this period. All participants gave written informed consent for enrollment. The study protocol was approved by the ethics committee of the Medical University of Vienna and complies with the Declaration of Helsinki. Data reporting was performed according to the STROBE and MOOSE guidelines.

### 2.2. Data Acquisition and Flow Cytometry

At the time of study enrollment, the patient characteristics were assessed; additionally, peripheral venous blood samples were taken and available for all study participants. All patients were enrolled in a stable condition free of any signs of congestion of acute cardiac ischemia. In patients presenting with ischemic HFrEF, the definition of heart failure was made at least 6 weeks after the acute ischemic event to overcome selection bias based on postinfarction myocardial stunning as recommended by the European Society of Cardiology [12, 13]. Routine laboratory parameters were analyzed and processed according to the local standards of the Department of Laboratory Medicine of the Medical University of Vienna. In addition, cells from fresh EDTA blood samples were stained with APC-Cy7-conjugated Anti-CD4 (BD Biosciences, San Jose, CA, USA) and FITC-conjugated Anti-CD8. Regulatory T cells were identified via their intracellular forkhead-box protein P3 (Fox-P3) and CD25 expression using PE-conjugated Anti-Fox-P3 (BioLegend, San Diego, CA, USA) as well as APC-conjugated Anti-CD25 (BioLegend, San Diego, CA, USA) in a second FACS panel. Stained cells were analyzed using a BD FACS Canto II Flow Cytometer System and FACSDiva software.

### 2.3. Follow-Up and Study Endpoints

The primary study endpoint was defined as cardiovascular mortality. The cause of death was evaluated by screening the national registry of death and revision of death certificated for the classification of cardiovascular mortality. Causes of death were specified according to the International Statistical Classification of Disease and Related Health Problem 10th revision (ICD-10). Cardiovascular mortality was determined as sudden cardiac death, fatal myocardial infarction, death after cardiovascular intervention, stroke, and causes of death effected from cardiac diseases.

### 2.4. Statistical Analysis

Categorical values were illustrated in counts and the respective percentage, continuous data as median and interquartile range (IQR). Categorical data are analyzed using the chi-square test, continuous data using the Kruskal-Wallis and Mann-Whitney *U* test. Cox regression hazard analysis was used to assess the influence of Tregs on survival. Accordingly, the influence of Tregs is presented as hazard ratio (HR) and the respective confidence interval (CI) which refers to an increase per one standard deviation (1-SD) in continuous values. To exclude all potential confounders, the multivariate model was adjusted for age, gender, and NT-proBNP. Moreover, survival curves of cardiovascular mortality were generated as the Kaplan-Meier plot. In statistical hypothesis, testing a *p* value of >0.05 (2-sided) was considered significant. A sample size of 100 patients (50 per group) was calculated to identify an assumed relative risk increase in mortality by 30% (power 80%, alpha 0.05). STATA 11 software (StataCorp LP, College Station, TX, USA) and PASW 18.0 (IBM SPSS, Armonk, NY, USA) were applied for statistical analysis.

The datasets gathered and analyzed during the current study are available from the corresponding author on reasonable request.

## 3. Results

### 3.1. Baseline Characteristics

A detailed description of the entire study population (*n* = 112) and stratified in iHFrEF (*n* = 57) and niHFrEF (*n* = 55) is illustrated in Table 1. In short, the average age was 65.6 years (IQR: 57.1-70.1) and 75% of participants were male. Comparing individuals with iHFrEF and niHFrEF, we observed a balanced cardiovascular risk profile in both groups, with regard to hypertension and type II diabetes mellitus. As expected, iHFrEF individuals presented with increased rates of hypercholesterolemia (iHFrEF: 68.4% vs. niHFrEF: 36.4%; *p* = 0.002) and coronary vessel disease (iHFrEF: 3.6% vs. niHFrEF: 100.0%; *p* < 0.001). Additionally, disease severity indicated via left ventricular ejection fraction (LVEF; *p* = 0.802) and N-terminal pro-Brain Natriuretic Peptide (Nt-proBNP; *p* = 0.790) were found to be comparable between types of HFrEF.

### 3.2. Distribution of T Cell Subsets

While the fraction of CD4^+^ (*p* = 0.080), CD3^+^ (*p* = 0.707), and CD8^+^ (*p* = 0.273) cells within T lymphocytes was comparable between iHFrEF and niHFrEF, a significantly higher fraction of Tregs was observed within the niHFrEF subgroup (iHFrEF: 0.42% vs. niHFrEF: 0.56%; *p* = 0.009; see Table 2).

### 3.3. Survival Analysis

#### 3.3.1. Cox Regression Analysis

After a mean follow-up time of 4.5 years, 32 (28.6%) patients died due to cardiovascular causes. We observed a significant inverse association of the fraction of Tregs with cardiovascular mortality in the entire study population with a crude HR per 1-SD of 0.53 (95% CI: 0.36-0.79; *p* = 0.002). Interestingly, while the predictive potential of Tregs was lost in the niHFrEF (crude HR per 1-SD of 0.41 (95% CI: 0.12-1.44, *p* = 0.163)) subgroup, its prognostic effect remained stable within the iHFrEF population (crude HR per 1-SD of 0.57 (95% CI: 0.36-0.96, *p* = 0.019)). Of note, the association remained stable even after comprehensive adjustment for potential confounders (adj. HR per 1-SD of 0.59 (95% CI: 0.36-0.96, *p* = 0.034), Table 3).

#### 3.3.2. Kaplan-Meier Survival Curves

**Figure 1:**
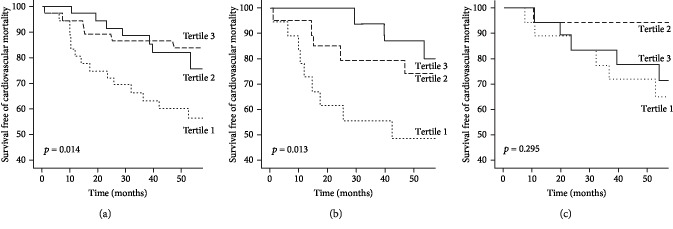
Survival curves of cardiovascular mortality. The Kaplan-Meier plots showing survival free of cardiovascular mortality in the total study collective (a) and patients stratified in ischemic HFrEF (b) as well as nonischemic HFrEF (c) according to tertiles of frequencies of regulatory T cells. Tertile 1 = low; tertile 2 = mid; tertile 3 = high.

The Kaplan-Meier survival curves according to tertiles of frequencies of Tregs were plotted and compared using the log-rank test in the total study collective, as well as patients stratified in iHFrEF and niHFrEF. Tertiles were stratified in 1 = low, 2 = mid, and 3 = high frequencies of Tregs. A period of 55 months was observed. The cardiovascular mortality event rate stratified by Treg fraction within the entire study population was 42.5% (low) vs. 24% (mid) vs. 16% (high) (*p* = 0.014; see Figure 1(a)). Moreover, a cardiovascular event rate of 52.5% (low) vs. 26% (mid) vs. 20% (high) (*p* = 0.013; see Figure 1(b)) was found in patients with iHFrEF. Patients with niHFrEF presented a cardiovascular mortality rate of 36% vs. 6% vs. 29% (tertile 1-3) (*p* = 0.295; see Figure 1(c)).

## 4. Discussion

To the best of our knowledge, the present investigation mirrors the first and largest in literature that highlighted a clear association of Tregs and patient outcome in HFrEF. Additionally, we were able to highlight that the observed effect of risk prediction is mainly attributed to ischemic etiology of HFrEF.

Recently, proinflammatory cytokines proved to have a pivotal role in the development and progression of HFrEF—however, less attention has been paid to the impact of cellular immunity. Shear stress and inflammatory mediators released by damaged myocardial tissue encourage sterile inflammation among myocytes and subsequently cardiac fibrosis [14] in HFrEF. Besides affecting the cardiac tissue itself, released cytokines proved to indirectly impact other organs as well [14, 15]. Moreover, the observation has been made that patients with HFrEF show elevated levels of inflammatory cytokines, such as IL-1*β* and IL-6 and TNF-alpha [16]. The latter correlates with disease severity, but generally, cytokines and their receptors seem to be a predictor of mortality in patients with progressed HFrEF [8, 16].

IL-1*β* promotes increased proliferation of monocytes in the spleen and monocytosis through the stimulation of stem cells in the bone marrow [17, 18]. Additionally, TNF-alpha modifies inflammation in the musculoskeletal system and adipose tissue [19, 20]. Both mentioned cytokines are also able to downregulate the expression of Ca^2+^ regulation gens in myocytes [21, 22]. This subsequently leads to a direct negative inotropic effect through disturbed intracellular Ca^2+^ homeostasis and therefore promotes cardiac remodeling, loss of LVEF, and tissue fibrosis in HFrEF [23, 24]. Furthermore, it has been illustrated that TNF-alpha and IL-1*β* are able to upregulate angiotensin II type 1 receptors on cardiac fibroblasts which additionally supports fibrosis [25, 26].

It is well established that the neurohumoral mechanism through the *β*-adrenergic nervous system and the renin angiotensin aldosterone system is trying to preserve the required cardiac function; however, this can lead to inflammation in other organs [27, 28]. Angiotensin II and catecholamine release that further stimulates the monocytopoiesis in the spleen and subsequently through long-term vasoconstriction skeletal muscle is harmed due to inadequate perfusion; thus, further inflammation is induced [29, 30]. Intestinal organs may suffer from underperfusion which potentially results in increased mucosal permeability and subsequently promotes systemic inflammation through translocation of bacteria and toxins into the blood stream [31–33]. Generally, inflammation leads to apoptosis of cardiomyocytes, myofibroblast differentiation, hypertrophy, endothelial dysfunction, and ultimately to myocardial remodeling and therefore dysfunction of the left ventricle [14, 15]. Thus, inflammatory processes, whether cardiac or systemic (e.g., obesity and rheumatoid arthritis), can trigger HFrEF through variable pathophysiological mechanisms.

Treg cells are the most important immune regulators and defined as CD4^+^CD25^+^Foxp3^+^ T cells. The intracellular transcription factor Foxp3 is a master regulator of Treg function and development and at present the most reliable molecular marker for Tregs. Moreover, it is vital for the identification of Tregs, as a T cell subset cannot be independently defined by their CD25 receptor surface expression [34].

This specific regulatory T cell population plays a pivotal role in fostering immune homeostasis and disruption of function or development is a primary cause of inflammatory and autoimmune diseases, e.g., rheumatoid arthritis. Tregs have been documented in inflammatory or damaged muscle tissue and atherosclerotic plaques [35, 36]. Through the secretion of anti-inflammatory cytokines such as TGF-b, IL-10, and IL-35, they prevent further progression of atherosclerosis and postinfarction inflammation [37–39]. In terms of myocardial infarction, genetic ablation of Foxp3^+^ Tregs leads to pronounced infiltration by proinflammatory T cells and thus severe cardiac inflammation and impaired cardiac function [40].

In addition, Tregs foster the healing process after myocardial infarction by modulating monocyte and macrophage differentiation [40]. The tissue regenerative effect of Tregs has been subject of emerging studies, but the specific mechanism of recruitment, and how they utilize regulatory effects within various local environments, remains yet unknown [41].

Our results further support the idea of cellular immunity in iHFrEF pathophysiology and corroborate Tregs as a predictor for long-term survival among iHFrEF patients. Higher levels of Tregs apparently contributed to a better overall survival. In our study collective, the niHFrEF subgroup showed both elevated levels of Tregs and better survival. In contrast, we found lower levels of Tregs and thus worse outcome within the iHFrEF subgroup. This observation supports previous findings [36], which documented reduced frequencies of circulating Tregs and also compromised function of Tregs in patients presenting with HFrEF. The latter, however, was not subject of the present study but would be interesting for further investigation.

Furthermore, the specific cause of the observed differences between the niHFrEF and iHFrEF subgroup remains unclear. A possible explanation for lower Treg counts in patients presenting with iHFrEF could be due to the inflammatory origin of the disease. iHFrEF is clearly related to atherosclerosis, which is driven by endothelial cell activation and plaque inflammation predominately mediated by Th1 cells [42]. In addition, an augmentation of the Th17/Treg ratio has been reported in ischemic heart tissue [43, 44]. The importance of this ratio has been discovered in other autoinflammatory diseases and could be related to plasticity of CD4^+^ T cell lineage differentiation [45]. Recently, the possibility of conversion to other phenotypes was discovered and lead to revision of the concept of terminally differentiated effector T cell lineage [46]. Thus, the influence of cell plasticity could have an important meaning in iHFrEF and would be an intriguing area for further investigation.

Previous research has also found decreased frequencies of Tregs in patients with nonischemic HFrEF [47]. However, the pathogenesis of niHFrEF is much more diverse than in iHFrEF due to its multifactorial causes, which are not limited to autoinflammatory etiology. Various other factors, for example, genetic predisposition or virus infections have been suggested as causes of niHFrEF. Moreover, no etiology can be found in the majority of cases and cardiomyopathy is considered idiopathic [48].

Overall, there is evidence for inflammatory processes in both etiologies of HFrEF, but the inflammatory component is apparently more dominant in iHFrEF due to its origin in atherosclerosis. Thus, it seems plausible that a lower frequency of Tregs is more closely associated with a worse outcome in the iHFrEF than in the niHFrEF subgroup. Cell plasticity and conversion to proinflammatory CD4^+^ cells could be a possible explanation for the lower Treg fraction in iHFrEF.

### 4.1. Limitations

The major limitation of the present study mirrors its small sample size. However, considering the performed methodology and flow cytometry, a satisfactory number of participants has been enrolled.

## 5. Conclusion

The frequency of Tregs in patients with iHFrEF was found to have a strong and inverse association with mortality in HFrEF. The present investigation supports the hypothesis that Tregs are an independent predictor of cardiovascular mortality in HFrEF patients. Most importantly, we extended the current knowledge by demonstrating that their prognostic potential is manly attributed via the predictive effect in iHFrEF while there was no effect observed in niHFrEF. Our results indicate a clear influence of Tregs in the pathogenesis and progression of HFrEF—especially in individuals presenting with iHFrEF, fostering the implication of cellular immunity in its pathophysiology. Tregs mirror an independent predictor for long-term survival among iHFrEF patients. Future investigations are needed to clarify the exact predictive mechanism of Tregs in HFrEF.

## Figures and Tables

**Table 1:** Baseline characteristics.

	Total collective	Ischemic HFrEF (*n* = 57)	Nonischemic HFrEF (*n* = 55)	*p* value
Age, years (IQR)	65.6 (57.1-70.7)	66.6 (57.7-70.8)	61.9 (56.4-70.7)	0.094
Male gender, *n* (%)	84 (75.7)	47 (82.5)	37 (69.1)	0.098
BMI, kg/m^2^ (IQR)	28.1 (24.6-31.3)	28.7 (25.3-31.9)	27.5 (24.1-30.8)	0.994
Diabetes mellitus, *n* (%)	42 (38.2)	23 (41.1)	19 (32.5)	0.478
Hypertension, *n* (%)	76 (68.5)	43 (75.4)	33 (61.8)	0.187
Current smoker, *n* (%)	20 (18.0)	11 (19.3)	9 (16.4)	0.682
Hypercholesterolemia, *n* (%)	59 (53.2)	39 (68.4)	20 (36.4)	**0.002**
Coronary vessel disease, *n* (%)	59 (53.2)	57 (100.0)	2 (3.6)	**<0.001**
Atrial fibrillation, *n* (%)	56 (50.5)	28 (49.1)	28 (51.9)	0.774
Left ventricular ejection faction				0.802
>40%, *n* (%)	27 (32.7)	18 (32.7)	19 (35.8)	
30-40%, *n* (%)	39 (36.4)	21 (38.2)	18 (34.0)	
<30%, *n* (%)	32 (29.9)	16 (29.1)	16 (30.2)	
Nt-proBNP, pg/ml (IQR)	1120 (443-2632)	1353 (449-3105)	851 (431-2117)	0.790
eGFR, ml/min/1.73m^2^ (IQR)	45.9 (35.0-54.7)	38.9 (32.2-50.2)	52.4 (41.9-59.9)	0.823
CRP, mg/dl (IQR)	0.36 (0.18-0.71)	0.32 (0.18-0.66)	0.37 (0.16-0.83)	0.520

Categorical data are presented as counts and percentages, continuous as median and IQR (interquartile range). Categorical data are analyzed using the chi-square test, continuous data using the Kruskal-Wallis test.

**Table 2:** Distribution of T cell subsets.

	Ischemic HFrEF	Nonischemic HFrEF	*p* value
Total lymphocytes (IQR)	2811 (2032-3753)	2986 (2260-4507)	0.183
% regulatory T cells within lymphocytes (IQR)	0.42 (0.30-0.68)	0.56 (0.39-0.80)	**0.009**
% CD4^+^ T cells within lymphocytes (IQR)	7.1 (5.2-10.4)	6.3 (4.2-8.3)	0.080
% CD3^+^ T cells within lymphocytes (IQR)	71.7 (67.6-76.6)	71.6 (65.8-77.5)	0.707
% CD8^+^ T cells within lymphocytes (IQR)	22.9 (17.7-34.2)	22.1 (14.1-32.3)	0.273

Continuous data are presented as median (interquartile range) and were compared between subgroups using the Mann-Whitney *U* test.

**Table 3:** Outcome analysis.

Cardiovascular mortality	Crude HR (95% CI)	*p* value	Adjusted HR (95% CI)	*p* value
Entire study cohort	0.53 (0.36-0.79)	**0.002**	0.60 (0.39-0.92)	**0.017**
Ischemic HFrEF	0.57 (0.35-0.91)	**0.019**	0.59 (0.36-0.96)	**0.034**
Nonischemic HFrEF	0.41 (0.12-1.44)	0.163	0.62 (0.17-2.31)	0.486

Cox proportional hazard model for % regulatory T cells within lymphocytes in patients with iHFrEF and niHFrEF. Hazard ratios (HR) for continuous variables refer to a 1-SD increase. The multivariate model was adjusted for age, gender, and NT-proBNP.

## Data Availability

The data used to support the findings of this study are available from the corresponding author upon request.
